# Alternatives to the in-person anaesthetist-led preoperative assessment in adults undergoing low-risk or intermediate-risk surgery: A scoping review

**DOI:** 10.1097/EJA.0000000000001815

**Published:** 2023-03-06

**Authors:** Philip Jonker, Sander van den Heuvel, Sanne Hoeks, Èmese Heijkoop, Robert-Jan Stolker, Jan-Wiebe Korstanje

**Affiliations:** From the Department of Anaesthesia, Erasmus MC University Medical Centre Rotterdam, Rotterdam, The Netherlands (PJ, SvdH, SH, EH, RJS, JWK)

## Abstract

**BACKGROUND:**

The design of the optimal preoperative evaluation is a much debated topic, with the anaesthetist-led in-person evaluation being most widely used. This approach is possibly leading to overuse of a valuable resource, especially in low-risk patients. Without compromising patient safety, we hypothesised that not all patients would require this type of elaborate evaluation.

**OBJECTIVE:**

The current scoping review aims to critically appraise the range and nature of the existing literature investigating alternatives to the anaesthetist-led preoperative evaluation and their impact on outcomes, to inform future knowledge translation and ultimately improve perioperative clinical practice.

**DESIGN:**

A scoping review of the available literature.

**DATA SOURCES:**

Embase, Medline, Web-of-Science, Cochrane Library and Google Scholar. No date restriction was used.

**ELIGIBILITY CRITERIA:**

Studies in patients scheduled for elective low-risk or intermediate-risk surgery, which compared anaesthetist-led in-person preoperative evaluation with non-anaesthetist-led preoperative evaluation or no outpatient evaluation. The focus was on outcomes, including surgical cancellation, perioperative complications, patient satisfaction and costs.

**RESULTS:**

Twenty-six studies with a total of 361 719 patients were included, reporting on various interventions: telephone evaluation, telemedicine evaluation, evaluation by questionnaire, surgeon-led evaluation, nurse-led evaluation, other types of evaluation and no evaluation up to the day of surgery. Most studies were conducted in the United States and were either pre/post or one group post-test-only studies, with only two randomised controlled trials. Studies differed largely in outcome measures and were of moderate quality overall.

**CONCLUSIONS:**

A number of alternatives to the anaesthetists-led in-person preoperative evaluation have already been researched: that is telephone evaluation, telemedicine evaluation, evaluation by questionnaire and nurse-led evaluation. However, more high-quality research is needed to assess viability in terms of intraoperative or early postoperative complications, surgical cancellation, costs, and patient satisfaction in the form of Patient-Reported Outcome Measures and Patient-Reported Experience Measures.

**KEY POINTS**

- Alternatives to the anaesthetist-led in-person preoperative assessment seem viable options.
- In the authors’ opinion, an elaborate digital questionnaire, evaluating the physical condition of all preoperative patients, could be a valuable method.
- High quality RCT's are needed to fill the knowledge gap and facilitate a base for a lasting, well structured transformation of preoperative evaluation.
- In future research, outcome measures should be standardised, e.g. same-day cancellation, complications, patient and provider satisfaction in the form of PREMS and PROMS, and costs.

## Introduction

In modern surgical practice, the preoperative evaluation is the starting point of safe, patient-centred perioperative care. By assessing patient risks of perioperative morbidity and mortality, patient's health can be optimised accordingly.

In the past 50 years the preoperative evaluation has changed from an ad-hoc evaluation to a full physical examination. The ad-hoc evaluation frequently resulted in same-day cancellations or higher perioperative risks if surgery was not postponed.^1^ Nowadays, in most countries, the preoperative evaluation is days to weeks before surgery and often led by an anaesthetist.^2,3^

This anaesthetist-led in-person preoperative evaluation is the contemporary standard of care worldwide, with a reported increase in perioperative safety.^4^ However, with increasing, but already high, degree of perioperative safety,^5^ and decreased operative risk^6^ along with increased use of comprehensive digital patient management systems, one wonders if there is any significant added value from an anaesthetist-led in-person preoperative evaluation in low-risk patients.

Moreover, it remains unclear whether alternative implementations of a preoperative evaluation such as telephone evaluation or evaluation by questionnaire would result in similar perioperative outcomes compared with an anaesthetist-led in-person preoperative evaluation. Furthermore, due to the current COVID-19 pandemic, alternatives to in-person consultation are widely used to ensure safety for patients and healthcare personnel. With the increasing number of surgical patients and restricted resources,^7^ we hypothesised that, without compromising patient safety, not all patients will require anaesthetist-led evaluation.

Since the data are limited and of heterogeneous nature, we decided to conduct a scoping review.^8^ The aim of this scoping review was to map the evidence in a systematic manner and identify gaps in research regarding alternative forms of preoperative evaluation compared with the anaesthetist-led in-person assessment.

## Methods

### Study design

The current scoping review assesses the literature of a personal preoperative evaluation by an anaesthetist compared with alternative forms of preoperative screening. The methodology of this review is based on the Preferred Reporting Items for Systematic Reviews and Meta-Analyses Extension for scoping reviews.^9^

### Search strategy

We developed an extensive literature search to identify publications regarding preoperative evaluation in the following online libraries: Embase, Medline, Web-of-Science, Cochrane Library and Google Scholar. No date restriction was used. After initial execution, our search was updated on the 22 October 2021. We identified any additional studies through hand-searches and reviewing the reference lists of the included articles. The full extensive search strategy can be found in the appendix (Appendix I).

### Eligibility criteria

For our primary inclusion, we used the Population, Concept, Context framework recommended by the Joanna Briggs Institute for scoping reviews (Table 1). We did not include studies that solely focused on the additive value of preoperative testing (e.g. ECG's, laboratory-testing or chest radiographs) as this was outside the scope of this review. Nevertheless, multiple large studies with high methodological quality^10–14^ have already concluded that a standardised ‘one-size-fits-all’ elaborate preoperative testing approach is inferior to personalised medicine.

**Table 1:** Population, Concept, Context layout – selection criteria for the inclusion of studies

Population	Adults, scheduled for noncardiac surgical intervention with general or regional anaesthesia
Concept	Comparison of: (1) Preoperative anaesthetic evaluation with an out-patient evaluation done by or under the responsibility of an anaesthetist or anaesthesia resident earlier than 24 h before surgery. With: (2) Preoperative anaesthetic evaluation, without an out-patient evaluation done by or under the responsibility of an anaesthetist or anaesthesia resident (3) No out-patient preoperative anaesthetic evaluation earlier than 24 h before surgery
Context	All countries, given their preoperative evaluation was in accordance with modern medicine guidelines - English or Dutch publication - Studies reporting on one or more of the following outcomes: surgical cancellation or delay; adverse events or mortality; patient satisfaction; perioperative management; costs

We excluded studies if their perioperative care was not comparable with standard clinical practice in modern medicine (i.e. studies in which large numbers of patients in remote areas were evaluated by a team of physicians once a year).

Studies were also excluded if full text was not available in English or Dutch or if their publication types were one of the following: case-reports, literature reviews, guidelines, meeting abstracts, abstract-only publications, comments, editorials, letters, textbooks or news.

Studies containing only patients undergoing high-risk procedures were also excluded, since the anaesthetist plays an undisputed key role in the preoperative optimisation of patients undergoing high-risk surgery, as stated in the 2014 European Society of Cardiology (ESC)/European Society of Anaesthesiology (ESA) Guidelines on non-cardiac surgery.^15^

### Study selection

Before evaluation, all duplicates were removed. All titles were separately screened for eligibility by two independent reviewers (PJ and SvdH) using EndNote version X9. If an article was potentially eligible based on the title, the abstract was evaluated.^16^ A third reviewer (JK) randomly assessed a sample of articles to determine that no eligible articles were missed. Disagreement between reviewers was resolved by discussion and if necessary, by the judgement of the third reviewer. Thereafter, full-text analysis was performed by both reviewers.

### Data extraction and analysis

Data extraction was performed by two independent reviewers. First, data were extracted by the first reviewer (PJ) and afterwards it was thoroughly checked by the second reviewer (SvdH). The following characteristics were extracted if available: year of publication, country of origin, type of study, population, age, ASA-PS, type of surgery, form of intervention, assignment to the different groups and primary and secondary outcomes of the study. When we report ‘surgical cancellation rates’, this represents cancellations within the 24-h period before surgery only, as opposed to earlier in the preoperative process. In some studies, all patients were first screened by an intervention (i.e. nurses or questionnaire) and afterwards by the control (the in-person screening by the anaesthetist). These studies are categorised as ‘one group post-test-only’ in this review.^17^ The type of surgery was divided into low, intermediate or high-risk surgery according to the 2014 ESC/ESA Guidelines.^15^

We extracted our main outcome measures using the reported outcomes by the studies included, if applicable and possible. This included calculating the sum of events when the intervention was used for multiple years, classifying surgical risk according to the ESC/ESA Guidelines if required and possible, and pooling patients’ characteristics if studies reported separate data per research arm only, that is mean age and American Society of Anesthesiologists physical status classification –ASA-PS. If the reporting of data was insufficient or unclear, the corresponding authors were contacted via e-mail.

### Quality assessment

Although quality assessment is not a prerequisite in scoping reviews, we nevertheless decided to review the included articles on methodological quality to complete the overview of the available literature. Three reviewers independently assessed the risk of bias and the quality of evidence of the included studies. For the randomised trial, the Cochrane Risk of Bias Assessment tool was used.^18^ For all other study types the NIH Study Quality Assessment Tools were used.^19^

## Results

### Selection process

**Fig. 1:**
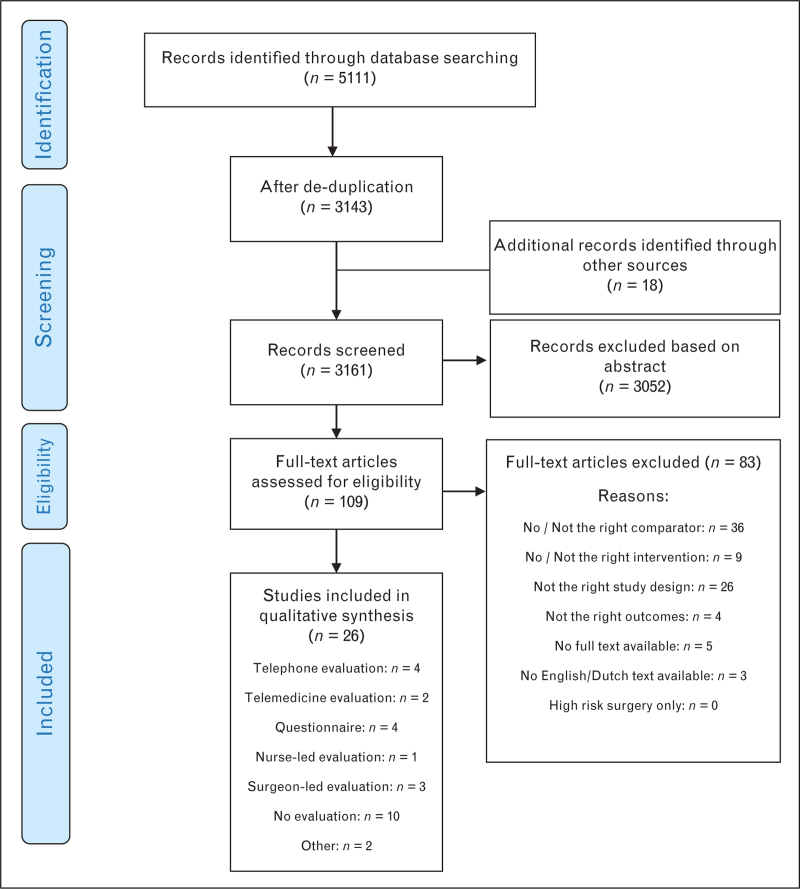
Preferred Reporting Items for Systematic Review and Network Meta-analyses flow diagram of study inclusion and exclusion.

We found a total of 5111 citations with our initial database search and another 18 through hand and reference searches and 26 of these were included in this review (Fig. 1). These reported on a variety of interventions: telephone evaluation, telemedicine evaluation, evaluation by questionnaire, nurse-led evaluation, surgeon-led evaluation, no evaluation until the day of surgery and other types of evaluation.

### Patient characteristics

In total, 361 719 patients were included in these studies, with study numbers varying from 84 to 157 410 patients. Mean age of patients varied between 34 and 75 years and patients were reported as ASA-PS 1 or 2 in 28 to 100% of cases. Most studies reported on both low and intermediate risk surgery (*n* = 8) and were conducted in North America (*n* = 11).

Study characteristics are summarised in Table 2, whereas study methods are summarised in Table 3.

**Table 2:** Characteristics of the studies included

Intervention	Article	Country of origin	Study type^a^	*N*	*N* (I)	*N* (C)	Mean age ± SD (years)	ASA-PS (I)	ASA-PS (C)	Surgical risk
Telephone evaluation	Subramanian, 2019^23^	United States	Pre/post	4966 (5640)^b^	3544^b^	2096^b^	NR	NR	NR	L
	Ming Teh, 2016^22^	Singapore	Pre/post	400	200	200	I: 35 ± 9 C: 34 ± 9	ASA 1: 56.5% ASA 2: 43.5%	NR	NR
	Ludbrook, 2015^21^	Australia	One group post-test only	193	–	–	58 ± 15	ASA 1: 14.9% ASA 2: 54.4% ASA 3: 29.7%	Same population	L, In
	Grant, 2012^20^	Australia	One group post-test only	514	–	–	50 ± 14	ASA 1: 14.6% ASA 2: 48.4% ASA 3: 19.6% ASA 4: 1.4% Unknown: 15.8%	Same population	L, In
Telemedicine evaluation	Kamdar, 2020^25^	United States	One group post-test only	2204 (3651)^b^	419 (645)^b^	1785 (3006)^b^	I: 56 ± 16 C: 61 ± 16	ASA 1: 8% ASA 2: 35.6% ASA 3: 40.6% ASA 4: 3.8% Unknown: 18.1%	ASA 1: 1.2% ASA 2: 27.1% ASA 3: 49.6% ASA 4: 4.3% Unknown: 17.9%	NR
	Applegate, 2013^24^	United States	RCT	155	78	77	I: 53 ± 15 C: 57 ± 15	ASA 1: 6.4% ASA 2: 50% ASA 3: 42.3% ASA 4: 1.3%	ASA 1: 2.6% ASA 2: 41.6% ASA 3: 51.9% ASA 4: 3.9%	In
Questionnaire	Mendes, 2013^27^	Brazil	One group post-test only	212	–	–	48 ± 16	ASA 1: 36.5% ASA 2: 55.4% ASA 3: 7.6% ASA 4: 0.5%	Same population	NR
	Reeves, 2003^28^	United States	One group post-test only	19 250	–	–	74 ± 8	NR	NR	L
	Lee, 1997^26^	Australia	Prospective	6130	4130	2 00	I: 40 ± 22 C: 58 ± 16	ASA 1: 63% ASA 2: 27% ASA 3: 9% ASA 4: 1%	ASA 1: 27% ASA 2: 47% ASA 3: 25% ASA 4: 2%	L, In
	Tompkins,1980^29^	United States	One group post-test only	84	–	–	NR	NR	NR	NR
Nurse-led	Van Klei, 2004^30^	The Netherlands	One group post-test only	4540	–	–	48 ± 16	ASA 1: 41.5% ASA 2: 52.5% ASA 3: 6.1%	ASA 1: 49.5% ASA 2: 45.0% ASA 3: 5.4% ASA 4: 0.1%	L, In
Surgeon-led	Power, 1999^31^	Australia	Pre/post	369	201	168	I: 50 ± 18 C: 48 ± 17	ASA 1: 39.9% ASA 2: 47.0% ASA 3: 13.1%	ASA 1: 46.3% ASA 2: 40.3% ASA 3: 13.4%	In
	Starsnic, 1997^33^	United States	Pre/post	3062	1519	1543	I: 45 ± 19 C: 47 ± 19	ASA 1: 39.4% ASA 2: 49.3% ASA 3: 11.0% ASA 4: 0.3%	ASA 1: 33.3% ASA 2: 55.2% ASA 3: 11.2% ASA 4: 0.2%	L, In
	Rutten, 1995^32^	The Netherlands	Pre/post	6407/600^c^	3122/ 300^c^	3258/300^c^	–	ASA 1: 55.0% ASA 2: 38.7% ASA 3: 6.3% ASA 4: 0%	ASA 1: 57.0% ASA 2: 35.7% ASA 3: 7.0% ASA 4: 0.3%	L, In
No evaluation^d^	Epstein, 2017^41^	United States	Retrospective	14 310 ± 252^e^	NR	NR	NR	ASA 1: 9.7% ± 0.5% ASA 2: 43.9% ± 0.7% ASA 3: 44.2% ± 0.7% ASA 4: 2.2% ± 0.2%	^f^	NR
	Blitz, 2016^4^	United States	Retrospective	64 418/27 928^g^	13 964^g^	13 964^g^	I: 48 ± 22 C: 49 ± 21	ASA 1: 23% ASA 2: 52% ASA 3: 19% ASA 4: 4% ASA 5: 0.03% Unknown: 2%	ASA 1: 20% ASA 2: 52% ASA 3: 23% ASA 4: 4% ASA 5: 0.03% Unknown: 2%	All
	Alboim, 2016^42^	Brazil	Retrospective	968	728	240	I: 70 (62 to 76) C: 73 (67 to 79)	ASA 1: 9.4% ASA 2: 83.3% ASA 3: 7.2% ASA 4: 0.1%	ASA 2: 65% ASA 3: 35%	L
	McKendrick, 2014^37^	United Kingdom	Pre/post	28 928	18 288	10 640	NR	NR	NR	L, In
	Knox, 2009^36^	Ireland	Pre/post	1390	721	669	NR	NR	NR	All
	Hariharan, 2006^35^	Trinidad	Prospective	213	129	84	NR	NR	NR	L, In
	Ferschl, 2005^34^	United States	Retrospective	6524	3729	2795	I: 33 ± 23^h^ C: 55 ± 18^h^	ASA 1: 27.1%^h^ ASA 2: 43.4%^h^ ASA 3: 12.5%^h^ ASA 4: 1.3%^h^ ASA 5: 0.1%^h^	ASA 1: 7.1%^h^ ASA 2: 49.8%^h^ ASA 3: 34.1%^h^ ASA 4: 2.2%^h^ ASA 5: 0%^h^	NR
	Mendes, 2005^38^	Brazil	Pre/post	60 693	17 082	43 611	NR	NR	NR	NR
	Van Klei, 2002^40^	The Netherlands	Pre/post	21 553 (24 685)^b^	14 148 (16 219)^b^	7405 (8466)^b^	I: 53 ± 18 C: 51 ± 18	NR	NR	All
	Pollard, 1999^39^	United States	Prospective	529	166	363	I: 58 ± 14 C: 59 ± 14	ASA 3 or 4: 33%	ASA 3 or 4: 31%	All
Other	Vazirani, 2012^44^	United States	Pre/post	5223	2565	2658	I: 61 ± 14 C: 64 ± 13	ASA 1: 3.3% ASA 2: 35.3% ASA 3: 58.1% ASA 4: 3.3% Unknown: 4.4%	ASA 1: 2.3% ASA 2: 35.3% ASA 3: 59.3% ASA 4: 3% Unknown: 4.6%	All
	Kinley, 2002^43^	United Kingdom	One group post-test only	1874	Nrs: 948 PHO: 926	1874	57	Nrs: ASA: 2.06 ± 0.75^i^ PHO: ASA 2.04 ± 0.75^i^	Same population	All

All, low + intermediate + high-risk surgery; C, control; I, intervention; In, intermediate risk surgery; L, low-risk surgery; NA, not applicable; NR, not reported; Nrs, nurse-led evaluation; PHO, pre-registration house officer; RCT, randomised controlled study.

a The terms prospective and retrospective refer to cohort studies, respectively.

b Numbers are amount of procedures.

c Random selection of 600 patients, with 300 in each group for classification of ASA-PS.

d No evaluation before 24 h prior to surgery.

e Mean number and SD per year for 11 consecutive years.

f The ASA-PS scores in Epstein were the scores of the entire population.

g Scores after propensity score matching.

h Calculated pooled mean and calculated pooled SD of both same day surgery suites and general operating rooms.

i ASA scores in Kinley are mean scores with standard deviation. Vazirani had hospitalist as intervention and Kinley had a combination of preregistration house officers and nurses as intervention.

**Table 3:** Summarised methods of the studies included – all control patients are communicated with in-person

Intervention	Article	SR	Communication (Int.)	Personnel (Int.)	Personnel (Crtl.)	Assignment to group	Study design
Telephone evaluation	Subramanian, 2019^23^^,^^a^	L	Telephone	Unknown	Anaesthetist	History and surgeon's choice	In the post-implementation period, all patients were screened by telephone. If indicated by history or deemed necessary by surgeon, patients were invited for in-person evaluation by anaesthetist. Others were solely screened by telephone
	Ming Teh, 2016^22^	–	Telephone	Nurses under supervision of anaesthetist	Anaesthetist	Time of evaluation	Assessment after implementation of telephone clinic, in comparison with the previous in-person evaluation by anaesthetist. High risk patients were later evaluated in-person, low risk patients were solely screened by telephone
	Ludbrook, 2015^21^	L, I	Telephone	Non-clinicians, computer aided	Anaesthetist	NA	All patients received a telephone screening preceding their clinical in-person evaluation by an anaesthetist. This was reviewed by both the procedural anaesthetist and a panel of senior anaesthetists
	Grant, 2012^20^	All	Telephone	Non-clinicians, computer aided	Anaesthetist	NA	All patients received a telephone screening preceding their clinical in-person evaluation by an anaesthetist. This was reviewed by a panel of anaesthetists
Telemedicine	Kamdar, 2020^25^	–	Telemedicine	Anaesthetist	Anaesthetist and anaesthesia resident	NA	Retrospective review after implementation of a telemedicine evaluation versus in person evaluation
	Applegate, 2013^24^	I	Telemedicine	Anaesthesia resident or NP	Anaesthesia resident or NP	Randomised	Random selection of patients for either telemedicine evaluation or in-person evaluation by an anaesthetist
Questionnaire	Mendes, 2013^27^	–	Questionnaire	None (computer only)	Anaesthetist	NA	Assessment of a pre-taken questionnaire compared with an in-person consultation by an anaesthetist blinded for the answers
	Reeves, 2003^28^	L	Questionnaire	The physician or other health professional	Anaesthetist	NA	Assessment of a pre-taken questionnaire compared with an in-person consultation by an anaesthetist
	Lee, 1997^26^	L, I	Questionnaire	Trained perioperative clerks and nurses	Anaesthetist	Questionnaire, surgeon's choice, choice patient, type of surgery	All patients were evaluated by questionnaire which decided whether they needed preoperative evaluation
	Tompkins,1980^29^	–	Questionnaire	None (computer only)	Anaesthetist	NA	All patients were first evaluated by a questionnaire and subsequently by the anaesthetist (blinded in half of the cases)
Nurse-led	Van Klei, 2004^30^	–	In-person	Nurses	Anaesthetist	NA	All patients were first evaluated by a nurse and subsequently by the anaesthetist
Surgeon-led	Power, 1999^31^	I	In-person	Surgeon	Anaesthetist	Time of evaluation	Evaluation of the implementation of an anaesthetist-led clinic in comparison to the previous existing surgeon-led clinic
	Starsnic, 1997^33^	L, I	In-person	Surgeon	Anaesthetist	Time of evaluation	Evaluation of the implementation of an anaesthetist-led clinic in comparison to the previous existing surgeon-led clinic
	Rutten, 1995^32^	L, I	In-person	Surgeon	Anaesthetist	Time of evaluation	Evaluation of the implementation of an anaesthetist-led clinic in comparison to the previous existing surgeon-led clinic
No evaluation	Epstein, 2017^41^	–	None	NA	NP or anaesthetist	Surgeon's choice	Comparison of patients evaluated in PEC by or under supervision of an anaesthetist versus no evaluation
	Blitz, 2016^4^	All	None	NA	One anaesthetist, one NP, three RNs, and one PCT	Screening tool	Comparison of patients evaluated in PEC by or under supervision of an anaesthetist versus no evaluation
	Alboim, 2016^42^	L	None	NA	Anaesthetist	Surgeon's choice	Comparison of patients evaluated in PEC by or under supervision of an anaesthetist versus no evaluation
	McKendrick, 2014^37^	L, I	None	NA	RN under supervision of anaesthetist	Time of evaluation	Evaluation of the effect of implementation of a PEC, compared to the previous period with no evaluation
	Knox, 2009^36^	All	None	NA	A doctor-led clinic under supervision of anaesthetist	Time of evaluation	Evaluation of the effect of implementation of a PEC, compared to the previous period with no evaluation
	Hariharan, 2006^35^	L, I	None	NA	Residents under supervision of anaesthetist	NR	Data analysis of attendance to the PEC and evaluation of its effect versus no evaluation
	Ferschl, 2005^34^	–	None	NA	Anaesthetist	Surgeon's choice	Comparison of patients evaluated in PEC by or under supervision of an anaesthetist versus no evaluation
	Mendes, 2005^38^^,^^b^	–	None	NA	Anaesthetist	Time of evaluation	Evaluation of the effect of implementation of a PEC, compared to the previous period with no evaluation
	Van Klei, 2002^40^	All	None	NA	Anaesthetist	Time of evaluation	Evaluation of the effect of implementation of a PEC, compared to the previous period with no evaluation
	Pollard, 1999^39^	All	None	NA	Specially trained RN, certified RN, anaesthetist, resident, or staff anaesthetist	Surgeon's operating schedule	Comparison between a preoperative evaluation 2 to 30 days versus within 24 h before surgery
Other	Vazirani, 2012^44^	L, I	In-person	Nurses under supervision of hospitalists	Mid-level providers under supervision of anaesthetist	Time of evaluation	Comparison of hospitalist-led PEC versus an anaesthetist-led PEC
	Kinley, 2002^43^	L, I	In-person	Preregistration house officers or RN	Anaesthetist	Randomised	Patients were assessed by either a nurse or a preregistration housing officer (PHO) and subsequently by an anaesthetist. Evaluations were compared

Ctrl, control; H, high; I, intermediate; Int, intervention; L, low; NP, nurse practitioner; PEC, preoperative evaluation clinic; RN, registered nurse; SR, surgical risk.

a Data is pooled from multiple years.

b Data from control is only for last 3 years, since the first year did not have complete data.

### Interventions

#### Telephone evaluation

Two one group post-test-only studies^20,21^ and two pre/post studies,^22,23^ which compared telephone evaluation to anaesthetist-led evaluation were included (Table 2). In the one group post-test-only studies,^20,21^ all patients were first evaluated by telephone before standard in-person evaluation by an anaesthetist (Table 3).

The two pre/post studies^22,23^ both showed significantly lower cancellation rates after implementation of the telephone evaluation when compared with the in-person evaluation (Table 4). Subramanian *et al.*^23^ showed an odds ratio (OR) of 0.80, 95% confidence interval (CI) 0.59 to 1.08, *P* *=* 0.148, in the first year and an OR of 0.62 (95% CI 0.44 to 0.88, *P* *=* 0.007) in the second year after implementation, with zero 30-day mortality in both groups during the study period. Ming Teh *et al.*^22^ showed zero cancellations in the intervention group and 4.5% cancellations in the control group (*P* *=* 0.01).

**Table 4:** Summarised outcomes of the studies included

Intervention	Article	SR	Surgical cancellations and delays	Adverse events and mortality	Patient satisfaction	Costs	Perioperative management
Telephone evaluation	Subramanian, 2019^23^^,^^a^	L	Intervention: 3.5% cancellations Control: 4.8% cancellations	Mortality: 0%	–	–	–
	Ming Teh, 2016^22^	–	Intervention: 0% cancellations Control: 4.5% cancellations	–	–	–	–
	Ludbrook, 2015^21^	L, I	–	–	–	Intervention: AUS $157 Control: AUS $295	–
	Grant, 2012^20^	All	–		–	–	89% could bypass in-person consult
Telemedicine	Kamdar, 2020^25^		Intervention: 2.96% cancellation rate Control: 3.23% cancellation rate	–	>90% satisfaction rate and preferred option in future with telemedicine	Estimated reduction of US $20 per visit (Q1 $15; Q3 $28).	–
	Applegate, 2013^24^	I	Intervention: 0% cancellations, 1,3% delays Control: 0% cancellations, 0% delays	–	High satisfaction rates in both groups	–	–
Questionnaire	Mendes, 2013^27^	–	–	–	–	–	68% could bypass in-person consult
	Reeves, 2003^28^	L	–	Perioperative events: 3.2% One week mortality rate: 0.02%	–	–	–
	Lee, 1997^26^	L, I	–	Intervention: 3% unanticipated adverse events Control: 6% unanticipated adverse events	–	–	–
	Tompkins, 1980^29^	–	–	–	66% satisfied, 24% neutral, 10% unsatisfied	–	In 93% more information via intervention vs control
Nurse-led	Van Klei, 2004^30^	–	Hypothetical increase of 0.3% in cancellations^b^	–	–	–	81% correctly evaluated as ready for surgery^c^ 6% correctly evaluated as not ready for surgery^c^ 13% not correctly evaluated^c^
Surgeon-led	Power, 1999^31^	I	–	–	–	Reduction of US $25.44 per patient Intervention: US $67.22 Control: US $41.78	Intervention: 3.3 tests per patient Control: 2.1 tests per patient
	Starsnic, 1997^33^	L, I	No case cancellations	–	–	Intervention: US $67.22 Control: US $41.78	–
	Rutten, 1995^32^	L, I	–	–	–	–	Significantly less lab tests, ECG, CXR, admissions and specialist consults in control vs intervention
No evaluation	Epstein, 2017^41^	–	First-case delay: 2.6 min per case less in control Turnover times: 7.5 min per case less in control	–	–	–	–
	Blitz, 2016^4^	All	–	Intervention: mortality rate of 0.2% Control: mortality rate of 0.1%	–	–	–
	Alboim, 2016^42^	L	–	No major adverse events or mortality Intervention: 34.0% minor adverse events Control: 29.7% minor adverse events	–	–	–
	McKendrick, 2014^37^	L, I	Intervention: 9.7% cancellations (3.1% medical) Control: 8.6% cancellations (2.0% medical)	–	–	–	–
	Knox, 2009^36^	All	Intervention: 38.3% cancellations (4.6% medical) Control: 15.8% cancellations (1.4% medical)	–	–	–	–
	Hariharan, 2006^35^	L, I	Intervention: 36% Control: 20%	–	–	–	–
	Ferschl, 2005^34^	–	Intervention: 15% cancellations Control: 6.6% cancellations	–	–	–	–
	Mendes, 2005^38^^,^^d^	–	Intervention: 35.2% cancellations (3.0% medical) Control: 29.6% cancellations (4% medical)^d^	–	–	–	–
	Van Klei, 2002^40^	All	Intervention: 6.3% cancellations (2.0% medical) Control: 4.6% cancellations (0% medical)	–	–	–	–
	Pollard, 1999^39^	All	Intervention: 13% cancellations Control: 13% cancellations	–	–	–	–
Other	Vazirani, 2012^44^	L, I	Intervention: 14.3% cancellations (4.9% medical) Control: 15.0% cancellations (8.5% medical)	Intervention: mortality rate of 0.4% inpatient surgeries Control: mortality rate of 1.3% inpatient surgeries	–	–	–
	Kinley, 2002^43^	L, I	–	–	–	–	Nurse: 13% insufficient assessment PHO: 15% insufficient assessment

CXR, chest-X-ray; H, high; I, intermediate; L, Low; PHO, preregistration house officers; SR, surgical risk.

a Data are pooled from multiple years.

b Only hypothetical since patients had standard evaluation as well.

c Nurse evaluation was compared with the standard in-person evaluation by an anaesthetist.

d Data from control is only for last 3 years, since the first year did not have complete data.

Both studies reporting on perioperative management^20,21^ concluded that the majority of patients were assessed adequately by telephone, with one study specifically stating that patients could bypass the anaesthetist-led evaluation.^21^ Only one study, Ludbrook *et al.*,^21^ reported on costs and the results show that the implementation of telephone evaluation comes with a reduction in costs.

#### Telemedicine evaluation

One randomised controlled trial (RCT)^24^ and one pre/post study,^25^ compared telemedicine evaluation to in-person evaluation, both performed by either a nurse practitioner or an anaesthesia resident in the first or second year of training. The RCT^24^ described one (1.3%) delay (of 1 h) in the telemedicine group *vs.* no delays in the in-person group and no cancellations in either group. The pre/post study^25^ described similar cancellation rates in the telemedicine group and the in-person group (3.0 *vs.* 3.3%, respectively). The authors also reported an estimated reduction in costs of US $20 per visit (IQR $15 to $28 USD) (Table 4). Both studies described high patient satisfaction.

#### Evaluation by questionnaire

Four studies compared a questionnaire with in-person evaluation.^26–29^ In three studies^27–29^ the questionnaire was added to the standard face-to-face assessment by an anaesthetist and in one study^26^ the questionnaire was used as a screening tool to decide if in-person evaluation was deemed necessary (Table 3).

One study^26^ reported a higher rate of unanticipated adverse events for patients in the in-person evaluation (risk ratio = 1.94, 95% CI: 1.42 to 2.64, *P* *<* 0.001) even after adjustment for confounders, while another study^28^ found a similar predictive value of co-morbidities identified by either the questionnaire or the in-person evaluation on adverse events or death.

There were high satisfaction rates with the computerised questionnaire evaluation.^29^ One study^27^ reported that in 67.9% of the patients screening by questionnaire was effective and no standard in-person assessment would have been necessary. One study^29^ stated that in the majority of patients the questionnaire scored higher in obtaining relevant information, (92.9%, *P* *<* 0.001), whereas another study^26^ stated that patients solely screened by questionnaire had a significantly higher risk of being inadequately prepared (3 *vs.* 5%; risk ratio = 1.61, 95% CI: 1.25 to 2.04, *P* *<* 0.001). The reasons for inadequate preparation included results of investigations not available, clinical history not fully documented, miscommunication, non-compliance with preoperative instructions and limited availability of interpreters for non-English-speaking patients.

#### Nurse-led evaluation

In this single one group post-test only study^30^ comparing a nurse-led evaluation with an anaesthetist-led evaluation, all patients were first evaluated by a nurse before all had the standard in-person assessment by an anaesthetist (Table 3). Patients cleared for surgery by the nurse without additional investigations, but not cleared by the anaesthetist were considered ‘cancelled’. The authors found a hypothetical increase of last-minute cancellations (0.3%). A similar assessment was found in 87% (95% CI: 86 to 88%) of cases; the nurses correctly classified 81% of patients as ‘ready’ without additional investigations, and 6% of patients as ‘not ready’ for surgery without additional investigations when compared with the anaesthetist evaluation (Table 4).

#### Surgeon-led evaluation

Three pre/post studies^31–33^ compared a surgeon-led preoperative clinic with the anaesthetist-led clinic (Tables 2 and 3). Starsnic *et al.*^33^ reported no case cancellations in either group and Power *et al.*^31^ reported a 37% reduction in costs. One study showed more tests per patient were used in the surgeon-led preoperative clinics compared with the anaesthetist-led clinics (3.3 vs. 2.1, *P* *<* 0.01)^31^ and another study showed significantly more laboratory tests, ECGs, or chest radiographs requested in the surgeon-led evaluation compared with the anaesthetist-led evaluation [90, 55 and 50% vs. 53, 43 and 10%, respectively (*P* *<* 0.05)].^32^

#### No evaluation

Ten of the studies included compared the anaesthetist-led preoperative evaluation to no outpatient evaluation until the day of surgery (Table 2).

Seven studies^34–40^ described cancellation rates, with six studies^34–38,40^ reporting a higher (15 *vs.* 6.6%,^34^ 36 *vs.* 20%,^35^ 38.3 *vs.* 15.8%,^36^ 9.7 *vs.* 8.6%,^37^ 35.2 *vs.* 29.6%,^38^ 6.3 *vs.* 4.6%,^40^) and one study^39^ a similar cancellation rate when no evaluation was compared with anaesthetist-led evaluation (Table 4). Four of the seven studies^36–38,40^ distinguished between cancellations for medical reasons and cancellations for other reasons (e.g. administrative), with three of these four studies reporting a higher medical cancellation rate in the no evaluation group (3.2 *vs.* 1.7%, *P* *<* 0.001;^36^ 4.6 *vs.* 1.4%, *P* *=* 0.013;^37^ 2.0 *vs.* 0.9%, *P* *<* 0.05^40^). Furthermore, Ferschl *et al.*^34^ reported a higher cancellation rate in patients with a higher ASA-PS (ASA-PS 1 3%; ASA-PS 2 4%; ASA-PS 3 5%; ASA-PS 4 14%, *P* *=* 0.06).

Two studies^34,41^ reported on surgical delays, with both concluding the anaesthetist-led clinic significantly reduced delays for the first scheduled case (*P* *<* 0.0001),^41^ the turnover times (*P* *<* 0.0001)^41^ and surgical delay (*P* *=* 0.015).^34^ Both studies reported that these delays were greater in patients with a higher ASA-PS.

Compared with the control group, one study found that the no assessment group had more adverse events (34.0 *vs.* 29.7%)^42^ and another study noted that the mortality rate was twice as high (risk ratio = 0.48, *P* *=* 0.04),^4^ but Alboim *et al.* found no mortality in either group.

#### Other interventions

Two studies described other types of interventions, one RCT^43^ and a pre/post study.^44^ In the RCT patients were randomised to be assessed by either by a pre-registration house officer without any special preoperative training or by a nurse, before all receiving standard in-person assessment by an anaesthetist (Table 3). Therefore, in our review, this study was identified as a one group post-test only study, since all patients were first evaluated by an intervention and afterwards by the control.

In the pre/post study,^44^ all patients were evaluated by mid-level providers under the supervision of either anaesthetists or hospitalists, after which both periods were compared. The pre/post study^44^ found a higher medical cancellation rate in the anaesthetist-led group versus the hospitalist group (8.5 *vs.* 4.5%, *P* *=* 0.065), (Table 4). The authors also described a significant reduction in mortality after introduction of the hospitalist-led evaluation (1.27 *vs.* 0.36%, *P* *=* 0.0158) and measured a significant increase in cardiac testing (3.4 *vs.* 1.5%, *P* *=* 0.012) and prescription of perioperative beta-blockers (33 *vs.* 26%, *P* *<* 0.001).

Kinley *et al.*^43^ reported that under assessment possibly affecting perioperative management was similar in both the group evaluated by nurses as the group evaluated by preregistration house officers (13 and 15%, *P* *>* 0.05). Under assessment was judged by one of two specialist registrars in anaesthesia. Whereas Vazirani *et al.* used the anaesthetist-led period for this.^44^

### Quality assessment

**Fig. 2:**
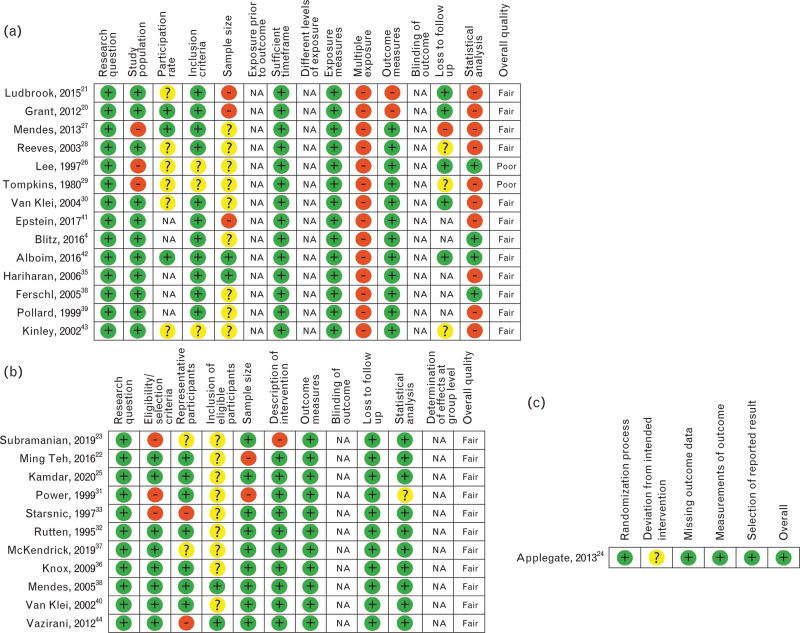
Quality assessment using the National Institute for Health National Heart, Lung and Blood Institute study quality assessment tools.^19^ (a) contains the quality assessment of cohort studies and one group post-test-only studies, (b) contains the quality assessment of pre/post studies and (c) contains the quality assessment of randomised controlled trials.

The quality assessment is shown in Fig. 2. Studies were of low-to-moderate quality overall, with only two RCT's (one of which^43^ was defined as a one group post-test only study in this review). The cohort studies and one group post-test only studies were mainly of moderate quality, with two studies^26,29^ being of poor quality (Fig. 2a). The pre/post studies were of moderate quality (Fig. 2b). The RCT showed a low risk of bias (Fig. 2c).

## Discussion

In this scoping review, we systematically mapped the literature comparing any preoperative evaluation other than an in-person evaluation by an anaesthetist – including no evaluation until the day of surgery – with an out-patient anaesthetist-led preoperative evaluation. We identified a total of 26 relevant articles, with a high level of heterogeneity between studies, types of intervention and outcome measures.

Most studies were conducted in the United States (40%) and included low-risk patients (28 to 100% of cases ASA-PS 1 or 2). The year of publication varied between 1980 and 2019, with all surgeon-led evaluation studies being published before 2000 and all telemedicine and telephone articles being published after 2010, therefore possibly influencing results and conclusions.

### Summary of key findings and recommendations for further research

It is important to state that the outcome measures, the healthcare systems in which the included studies were conducted, as well as the year of publication were highly heterogeneous between the studies, making it difficult to draw generalisable conclusions. In particular, the studies showed a wide variation in the design of patient evaluation: whether patients had been screened by nurses or other practitioners before the anaesthetic assessment; whether the intervention was either added to the anaesthetic assessment or was a completely separate assessment; and whether the evaluating anaesthetists were blinded to the previous assessments. Since these variations have a high probability of influencing study outcomes, this heterogeneity is a relevant finding of our review.

Furthermore, the number of studies available concerning the effectiveness of an anaesthetist-led in-person evaluation in its entirety is limited and of low-to-moderate quality, with only two RCTs and a high number of publication dates before 2010.

Moreover, in some of the included studies,^4,22,23,26,34,41,42^ the indication for either an in-person evaluation by the anaesthetist or one of the interventions, was made by the surgeon or an assessment tool, making the intervention and control groups less comparable and introducing bias by indication.

Nevertheless, the included studies consistently showed similar results, suggesting telephone evaluation, telemedicine evaluation or evaluation by questionnaire could be, in certain cases, viable alternatives for in-person preoperative screening by an anaesthetist. There was no increase in surgical cancellation or surgical delay found for both telephone and telemedicine evaluation.^22–25^ For the telemedicine evaluation and questionnaire,^24,25,29^ satisfaction rates were high, and two studies concluded the questionnaire added information to the consultation.^27,29^ One study reporting on telephone evaluation ^21^ showed a reduction in costs compared with the anaesthetist-led evaluation, which was explained by the time needed to assess the patient.

Morbidity and mortality were low in all studies reporting these outcomes.^4,23,26,28,42,44^ This creates possibilities for future research without compromising patient safety. However, it should be noted that these results pertain to (very) low-risk surgery (i.e. cataract surgery) as well, where few complications are expected in general. Significantly, the study conducted by Lee *et al.*^26^ describing the evaluation by questionnaire concluded that there were more adverse events in the anaesthetist-led group, even after adjustment for risk factors. However, indication bias is likely to play a role since the assignment to the group was based on the surgeon's decision and this resulted in the sickest patients being sent to the anaesthesiology department for in-person evaluation, creating large baseline differences between the groups.

The results of our study correspond to the findings of systematic reviews about telephone evaluation^45^ and telemedicine,^46^ concluding these are safe and appropriate alternatives for preoperative evaluation.

Another surprising finding was one study^44^ reporting a decrease in both mortality rate and cancellation rate in the hospitalist-led clinic when compared with the anaesthetist-led evaluation. However, evaluation was carried out solely by mid-level providers with anaesthetists and hospitalists having a supervisory role. Furthermore, the intervention was implemented after extensive training of preoperative clinic personnel, possibly influencing the outcome. Lastly, since it was carried out in a veterans hospital, there is a chance of selection bias.

In contrast, surgeon-led evaluation is reported to be not as adequate as the anaesthetist-led evaluation^31,32^ and seems to increase costs.^31,33^ However, all studies reporting on surgeon-led evaluation are published before the year 2000 and thereby may be less applicable to current situations.

Moreover, no evaluation until the day of surgery is likely to increase same-day cancellations,^34–41^ and seems to increase morbidity and mortality,^4,42^ which seems a logical finding.

The current review indicates multiple focus points for future research considering alternative forms of preoperative assessment. Regarding study design, there is a need for high-quality research. RCTs have a low risk of bias and are the preferred design, however they demand time and resources, and it may be challenging to include large groups of patients. In contrast, prospective interventional studies (i.e. pre/post stepped-wedge studies) may be feasible at a larger scale, however correction for case-mix bias will be essential.

Considering all the issues, we recommend non-inferiority trials that contain two individual study arms: one arm evaluating in-person preoperative screening by an anaesthetist and one arm evaluating alternative methods. Since high-risk patients are assumed to still benefit from in-person consultation, a triage algorithm should be developed to identify these patients. Such a triage algorithm can be studied alongside current practice, and close monitoring should take place when being used in a randomised trial. All patients should be evaluated simultaneously, thus resulting in a reduced risk of confounding factors, and assignment to the study arm should be randomised.

Such a well-structured study would create the possibility of comparing all different methods properly and to state definite outcome measures (i.e. intraoperative or early postoperative complications, mortality, surgical cancellation, costs, length of stay and number of admissions) considering the different methods of preoperative screening, a factor lacking in the included studies in this review.

Most of the included studies reported on surgical cancellation rates, with only six studies describing perioperative complications and mortality.^4,23,26,28,42,44^ We recommend future researchers focus on a core set of outcomes such as intraoperative or early postoperative complications, mortality, surgical delay or cancellation and total cost reduction. Costs were measured in only four of the studies included. However, with a cost-restrained future in medicine, cost reduction strategies will be critical drivers for change in future healthcare and should be a point of focus.

Another outcome which was not researched well was patient satisfaction in terms of Patient-Reported Evaluation Measures (PREMs) or Patient-Reported Outcome Measures (PROMs). However, to measure these outcomes, a structured approach should be used, where patients receive a systematic questionnaire and declare their satisfaction based on a predetermined scale.^24^

Regarding the design of the intervention, the optimal alternative is open for debate. In our opinion, an elaborate digital questionnaire, evaluating the physical condition of all patients preoperatively could be a valuable method. Although none of the included studies about evaluation by questionnaire reported on costs, a preoperative (electronic) questionnaire seems a cost-effective and time efficient alternative. This questionnaire can be filled out at home or after the surgical assessment, saving time and costs for both the patient and the medical specialist, and reducing face-to-face contact, which is beneficial considering the COVID-19 pandemic. Moreover, the COVID-19 pandemic has made remote access and digitisation of information more commonplace. Therefore, we expect both patients and physicians to have an even more positive attitude towards digital consultation than portrayed in the studies included in this review, which predate COVID.

When certain red-flag-questions in the questionnaire are answered positively, the patient will be invited to an in-person assessment where physical examination, additional testing, and referral to other specialists are among the options. In this way, time and resources are used optimally, making healthcare more efficient.

In summary, future research should focus on a high-quality study design with a standardised set of outcome measures, and above all on the needs and demands of the healthcare system in which the study is conducted.

### Limitations

The current review has several limitations, most of which are inherent with scoping reviews. First, as mentioned before, the available studies are of low-to-moderate quality, with a small number of RCTs, and many studies have a potential risk of bias.

Moreover, we chose to exclude studies reporting on high-risk surgery, since current guidelines^15^^,^^47^ recommend patient optimisation and elaborate testing before these procedures, and these need a physical consultation. This exclusion inevitably limits generalisability and probably contributes to the low morbidity and mortality rates.

Finally, as stated before, outcome measurements differed significantly between studies, thus creating difficulty in comparing these publications.

## Conclusion

Our scoping review found that a limited number of studies have been published comparing an anaesthetist-led in person preoperative assessment with other types of preoperative evaluation, and that there was a high level of heterogeneity between these studies. Moreover, the methodological quality of the included studies was low to moderate at best.

However, these studies do show that alternatives to the anaesthetist-led in person preoperative assessment are viable. Therefore, high-quality studies, preferably RCTs, are necessary to make valuable recommendations regarding future clinical practice. Furthermore, a set of uniform outcome measures should be created to facilitate comparison between different types of preoperative assessments. Such outcomes should at least include intraoperative or early postoperative complications, mortality, surgical delay or cancellation, patient and anaesthetist satisfaction including PREMs and PROMs, and costs.

## Supplementary Material

Supplemental Digital Content
