# Supplementary material for: Alternatives to the in-person anaesthetist-led preoperative assessment in adults undergoing low-risk or intermediate-risk surgery: A scoping review
Source: Eur J Anaesthesiol. 2023 Mar 6;40(5):343–55. doi: 10.1097/EJA.0000000000001815 (PMC10097490; doi:10.1097/EJA.0000000000001815)
Supplement: Supplemental Digital Content [file ejanet-40-343-s001.docx]

20211022 (Update) Philip Jonker

Effectiviteit preoperatieve screening polikliniek laag-risico patiënten

| **Database searched** | **via** | **Years of coverage** | **Records** | **Records after duplicates removed** |
| --- | --- | --- | --- | --- |
| Embase | Embase.com | 1971 - Present | 2177 | 2148 |
| Medline ALL | Ovid | 1946 - Present | 1681 | 750 |
| Web of Science Core Collection | Web of Knowledge | 1975 - Present | 915 | 138 |
| Cochrane Central Register of Controlled Trials | Wiley | 1992 - Present | 138 | 34 |
| Other sources: Google Scholar (200 top-ranked) | | | 200 | 73 |
| **Total** | | | **5111** | **3143** |

**Embase.com**

('preoperative evaluation'/mj/de OR (('physical examination'/mj/exp OR 'anamnesis'/mj/exp OR 'medical history'/mj/de OR 'blood examination'/mj/de OR 'laboratory test'/mj/exp OR 'electrocardiogram'/mj/de OR 'lung function test'/mj/de) AND ('preoperative care'/mj/de OR 'preoperative period'/mj/exp)) OR (((preop* OR pre-op* OR preanest* OR pre-anest* OR preanaest* OR pre-anaest* OR preadmission* OR pre-admission*) NEAR/3 (eval* OR assess* OR physical-assess* OR clearance* OR screening OR physical-exam* OR medical-histor* OR medical-evaluat* OR history-tak* OR medical-interview* OR patient-histor* OR ECG* OR cardiogram* OR EKG* OR electrocardiogram* OR electrocardiograph* OR interview* OR test* OR examinat* OR visit* OR risk-stratif*)) OR ((prior OR before) NEAR/6 surg* NEAR/6 (eval* OR assess* OR clearance* OR screening OR physical-exam* OR medical-histor* OR history-tak* OR medical-interview* OR patient-histor* OR ECG* OR cardiogram* OR EKG* OR electrocardiogram* OR electrocardiograph* OR interview*))):ti) AND ('anesthesist'/exp OR telephone/exp OR anesthesia/exp OR telemedicine/exp OR questionnaire/exp OR interview/exp OR (anesthe* OR anaesthe* OR preanesthe* OR preanaesthe* OR interview* OR telemedicine OR phone* OR telephone* OR questionnaire* OR surgeon-led OR nurse-led OR nurse-practitioner-led):ab,ti,kw) NOT (juvenile/exp NOT adult/exp) NOT ([Conference Abstract]/lim) AND ([ENGLISH]/lim OR [Dutch]/lim)

**Medline ALL Ovid**

(((*Medical History Taking/ OR exp *Physical Examination/ OR exp *Clinical Laboratory Techniques/ OR exp *Electrocardiography/ OR exp *Respiratory Function Tests/ OR *Hematologic Tests/) AND (exp *Preoperative Care/ OR *Preoperative Period/)) OR (((preop* OR pre-op* OR prean* OR pre-an*) ADJ3 (eval* OR assess* OR clearance* OR screening OR physical-exam* OR medical-histor* OR history-tak* OR medical-interview* OR patient-histor* OR ECG* OR cardiogram* OR EKG* OR electrocardiogram* OR electrocardiograph* OR interview* OR test*)) OR ((prior OR before) ADJ6 (eval* OR assess* OR clearance* OR screening OR physical-exam* OR medical-histor* OR history-tak* OR medical-interview* OR patient-histor* OR ECG* OR cardiogram* OR EKG* OR electrocardiogram* OR electrocardiograph* OR interview*))).ti.) AND (exp Telephone/ OR exp Anesthesia/ OR exp Telemedicine/ OR "Surveys and Questionnaires"/ OR Interviews as Topic/ OR (anesthe* OR anaesthe* OR preanesthe* OR preanaesthe* OR interview* OR telemedicine OR phone* OR telephone* OR questionnaire* OR surgeon-led OR nurse-led OR nurse-practitioner-led).ab,ti,kf.) NOT (Adolescent/ OR exp Child/ OR exp Infant/ NOT Adult/) AND (English OR dutch).lg NOT (news OR congres* OR abstract* OR book* OR chapter* OR dissertation abstract*).pt.

**Web-of-Science**

((TI=(((preop* OR pre-op* OR preanest* OR pre-anest* OR preanaest* OR pre-anaest* OR preadmission* OR pre-admission*) NEAR/2 (eval* OR assess* OR physical-assess* OR clearance* OR screening OR physical-exam* OR medical-histor* OR medical-evaluat* OR history-tak* OR medical-interview* OR patient-histor* OR ECG* OR cardiogram* OR EKG* OR electrocardiogram* OR electrocardiograph* OR interview* OR test* OR examinat* OR visit* OR risk-stratif*)) OR ((prior OR before) NEAR/5 surg* NEAR/5 (eval* OR assess* OR clearance* OR screening OR physical-exam* OR medical-histor* OR history-tak* OR medical-interview* OR patient-histor* OR ECG* OR cardiogram* OR EKG* OR electrocardiogram* OR electrocardiograph* OR interview*)))) AND TS=((anesthe* OR anaesthe* OR preanesthe* OR preanaesthe* OR interview* OR telemedicine OR phone* OR telephone* OR questionnaire* OR surgeon-led OR nurse-led OR nurse-practitioner-led))) AND DT=(Article OR Review OR Letter OR Early Access) AND LA=(English)

**Cochrane**

((((preop* OR pre NEXT op* OR preanest* OR pre NEXT anest* OR preanaest* OR pre NEXT anaest* OR preadmission* OR pre NEXT admission*) NEAR/3 (eval* OR assess* OR physical NEXT assess* OR clearance* OR screening OR physical NEXT exam* OR medical NEXT histor* OR medical NEXT evaluat* OR history NEXT tak* OR medical NEXT interview* OR patient NEXT histor* OR ECG* OR cardiogram* OR EKG* OR electrocardiogram* OR electrocardiograph* OR interview* OR test* OR examinat* OR visit* OR risk NEXT stratif*)) OR ((prior OR before) NEAR/6 surg* NEAR/6 (eval* OR assess* OR clearance* OR screening OR physical NEXT exam* OR medical NEXT histor* OR history NEXT tak* OR medical NEXT interview* OR patient NEXT histor* OR ECG* OR cardiogram* OR EKG* OR electrocardiogram* OR electrocardiograph* OR interview*))):ti) AND ((anesthe* OR anaesthe* OR preanesthe* OR preanaesthe* OR interview* OR telemedicine OR phone* OR telephone* OR questionnaire* OR surgeon NEXT led OR nurse NEXT led OR nurse NEXT practitioner NEXT led):ab,ti,kw)

**Google Scholar**

"preoperative|preanesthesia|preanesthetic evaluation|assessment|clearance|screening" anesthesist|anaesthesist|anesthesists|anaesthesists|interview|telemedicine|telephone|questionnaire|"surgeon|nurse|practitioner led"
